# Identification of genes differentially expressed in T cells following stimulation with the chemokines CXCL12 and CXCL10

**DOI:** 10.1186/1471-2172-5-17

**Published:** 2004-08-05

**Authors:** JE Nagel, RJ Smith, L Shaw, D Bertak, VD Dixit, EM Schaffer, DD Taub

**Affiliations:** 1 Clinical Immunology Section, Laboratory of Immunology, Gerontology Research Center, National Institute on Aging, NIH, 5600 Nathan Shock Dr., Baltimore, MD 21224 USA

## Abstract

**Background:**

Chemokines are involved in many biological activities ranging from leukocyte differentiation to neuronal morphogenesis. Despite numerous reports describing chemokine function, little is known about the molecular changes induced by cytokines.

**Methods:**

We have isolated and identified by differential display analysis 182 differentially expressed cDNAs from CXCR3-transfected Jurkat T cells following treatment with CXCL12 or CXCL10. These chemokine-modulated genes were further verified using quantitative RT-PCR and Western blot analysis.

**Results:**

One hundred and forty-six of the cDNAs were successfully cloned, sequenced, and identified by BLAST. Following removal of redundant and non-informative clones, seventeen mRNAs were found to be differentially expressed post treatment with either chemokine ligand with several representing known genes with established functions. Twenty-one genes were upregulated in these transfected Jurkat cells following both CXCL12 and CXCL10, four genes displayed a discordant response and seven genes were downregulated upon treatment with either chemokine. Identified genes include geminin (GEM), thioredoxin (TXN), DEAD/H box polypeptide 1 (DDX1), growth hormone inducible transmembrane protein (GHITM), and transcription elongation regulator 1 (TCERG1). Subsequent analysis of several of these genes using semi-quantitative PCR and western blot analysis confirmed their differential expression post ligand treatment.

**Conclusions:**

Together, these results provide insight into chemokine-induced gene activation and identify potentially novel functions for known genes in chemokine biology.

## Background

CXC and CC chemokines are small soluble proteins expressed and secreted by a number of cell types during the initial host response to injury, allergens, antigens, or invading microorganisms [1]. These ligands selectively attract leukocytes to inflammatory foci via facilitation of cellular adhesion, transendothelial migration, chemotaxis and cellular activation. Receptors for chemokines are members of the large family of G-protein receptors that signal via heterotrimeric guanine nucleotide-binding proteins of the Gαi-subclass [2]. Chemokine receptors can be subdivided into specific families based on their specificity for C, CC, CXC, or CX3C chemokine ligands. Three distinct types of receptor binding are currently recognized: (1) chemokine receptors that bind only one chemokine specific ligand; (2) chemokine receptors that bind more than one chemokine often with different binding affinities; and (3) promiscuous chemokine receptors that bind to numerous chemokines [2].

The chemokine receptor CXCR4 binds to the CXC chemokine, CXCL12 and functions as a co-receptor for HIV-1 [3]. CXCR4 is broadly expressed by many cells within the body including cells of the immune and the central nervous system [4-7]. This receptor mediates the migration of resting leukocytes and hematopoietic progenitors in response to its specific ligand [8,9]. CXCL12-induced chemotaxis is inhibited by pertussis toxin, enhanced *in vitro *by IL-3, and selectively inhibited by soluble ephrin-B receptor. [10]. In addition, proinflammatory stimuli such as lipopolysaccharide, tumor necrosis factor (TNF-α) or interleukin-1 potentiates lymphocyte-and monocyte-, but not neutrophil-mediated CXCL12 responses [11,12]. Furthermore, CXCL12 is an extremely potent *in vitro *and *in vivo *chemoattractant for mononuclear cells and lymphocytes [13]. CXCL12 is expressed in the cells forming Hassall's corpuscles and plays a significant role in the elimination of apoptotic thymocytes in normal and HIV-1-infected thymic tissues [14]. In addition to the bone marrow, quantitative PCR analysis has detected expression of CXCL12 in the lymph nodes, lung, and liver [15]. Autocrine and paracrine production of CXCL12 by peripheral blood CD34^+^CD38^+ ^cells also appears to trigger their transition from G_0 _to G_1 _and, in conjunction with thrombopoietin, enhances their survival through signal transduction mediated by the PI3K/AKT proteins [16]. Together these data support a role for CXCL12 as a critical factor for cellular growth and differentiation, cellular trafficking, myelopoiesis, and organ vascularization [17,18].

In contrast to CXCL12, considerably less is known about the chemokine CXCL10. CXCR3 (GPR9; CD183), the receptor for CXCL10 also binds the CXC chemokines CXCL9 and CXCL11 [19]. Recent studies of the CNS have suggested that CXCR3 additionally binds CCL21 [20]. CXCL10 is secreted by a variety of cell types, including monocytes, endothelial cells, fibroblasts, and astrocytes. CXCL10 is also a chemoattractant for human monocytes, natural killer and T cells (preferentially Th1 cells), and appears to modulate adhesion molecule expression and function [21-23]. CXCL10 is expressed in keratinocytes, lymphocytes, monocytes, and endothelial cells during Th1-type inflammatory diseases such as psoriasis and atopic dermatitis, but only at very low basal levels in normal keratinocytes [24,25]. CXCL10 inhibits bone marrow colony formation by CD34^+ ^cells in the presence of stem cell growth factor (SCGF), colony stimulating factor 2 (granulocyte-macrophage) (CSF2; GM-CSF), or a combination of SCGF and erythropoietin (EPO). Moreover, CXCL10 has antitumor activity *in vivo *and is a potent inhibitor of angiogenesis [26]. This antitumor activity appears to be mediated by the ability of CXCL10 to recruit lymphocytes, neutrophils, and monocytes into inflammatory infiltrates. Moreover, CXCL10 has also been recently shown to be a Ras target gene and is overexpressed by a number of colorectal cancers [27]. Overall, CXCL10 is an important chemokine for mediating delayed-type hypersensitivity responses and a potent regulator of colony formation, angiogenesis, adhesion and cell migration.

Alterations in gene expression are important determinants of cellular physiology. As a consequence, the identification, cloning and characterization of differentially expressed genes can provide relevant and important insights into a variety of biological processes. To investigate and compare the similar and distinct genes induced by the chemokines, CXCL12 and CXCL10, in normal physiology, we utilized differential display analysis to identify mRNAs in a Jurkat T cell line expressing endogenous CXCR4 and transfected with human CXCR3 gene. We have identified and cloned several differentially expressed genes displaying both elevated and diminished expression in the context of specific chemokine receptor ligation. The possible relevance of such differential responses within normal immune responses and in normal T-cell physiology is discussed.

## Methods

### Differential display

Total RNA was isolated using the Qiagen RNeasy^® ^kit (Qiagen Inc., Valencia, CA) and treated with DNase I (GenHunter, Nashville, TN). Two micrograms of the total RNA was derived from subclone of CXCR3-transfected Jurkat T cells (generously donated by Dr. Thomas Hamilton, Lerner Research Institute, Cleveland, OH) cultured for 24 h in the presence and absence of 1 μg/ml of bioactive CXCL12 or CXCL10 (PeproTech). It should be noted that the CXCR3-transfected Jurkat T cells were subcloned from the original cultures. Subclones of the CXCR3-transfected lines were initially generated at the initiation of these studies so that homogenous CXCR3-bearing cells were available. A single Jurkat subclone was selected and examined for coexpression of both CXCR3 and CXCR4 by flow cytometry (Table 1). The isolated RNAs were subsequently reverse-transcribed with 400 units of MMLV reverse transcriptase (GenHunter) in three separate reactions each containing 2 uM of a one-base-anchored H-T_11_M (i.e. H-T_11_G, H-T_11_A and H-T_11_C) primer (RNAimage^®^, GenHunter) and 20 uM dNTP for 60 min at 37°C. After heat inactivation of the reverse transcriptase at 75°C for 5 min, 2 μl of each reverse transcription reaction was added to 18 μl of a PCR master mix containing 2 uM of an H-T_11_-arbitrary primer, 1 U Taq polymerase (Qiagen), 2 uM dNTP, and a-[^33^P]dATP. Each primer pair was denatured at 94°C for 30 sec, annealed at 40°C for 2 minutes and extended at 72°C for 30 sec for 40 cycles with a final extension for 10 minutes. [^33^P]-labeled PCR products were resolved on a 6% denaturing polyacrylamide gel. The autoradiogram was inspected on a light box and differentially expressed bands marked by needle punches. The punched film was carefully oriented on the dried gel and the marked bands excised with a scalpel blade. Glycogen (10 mg/ml), 3 M sodium acetate, and 85% EtOH were added and after overnight storage at -80°C, the DNA was precipitated by centrifugation. Each DNA was subsequently reamplified using the same PCR primer set and conditions except that the dNTP concentration was increased to 20 uM and no isotope was added to the mixture. Reamplified PCR products were resolved on a 1.5% AmpliSize™ (BioRad, Richmond, CA) agarose gel, stained with SYBR^® ^Gold (Molecular Probes, Eugene, OR) and extracted from the gel using a QIAEX II kit (Qiagen). Successfully amplified bands were cloned using the PCR-TRAP^® ^cloning vector system (GenHunter) and ligated into GH-competent cells. Following transformation, only clones that contain an insert are capable of growing on LB-Tet agarose plates. The cloned insert was subsequently checked by colony-PCR using primers flanking the PCR-TRAP^® ^vector and the insert sequenced to identify genes differentially expressed between control and chemokine-treated CXCR3-transfected Jurkat T cells.

**Table 1:** Flow cytometric analysis of transfected Jurkat T cell lines

% Positive (MFI)				
Cell Line	CD3	CXCR4	CXCR3	CXCR2
Jurkat-Neo	98 (138)	99 (118)	2 (5)	4 (4)
Jurkat-CXCR3	98 (154)	99 (124)	99 (24)	5 (8)
Jurkat-Bcl2	96 (144)	96 (124)	5 (6)	7 (5)

CXCR3-, Bcl2-and Neo-transfected Jurkat T cells were stained using IgG FITC-labeled antibodies to CD3, CXCR4, CXCR3, and CXCR2 and analyzed for cell surface protein expression via flow cytometric analysis. The data are expressed as % positive (mean fluorescence intensity). It should be noted that the CXCR3-transfected Jurkat cells expressed low levels of CXCR3 on their cells surface suggesting a lower receptor density in comparison to CXCR4.

### Quantitative analysis of PCR fragments

One microgram of DNase I-treated total RNA from control, CXCL12-or CXCL10-treated CXCR3-transfected Jurkat T cells was reverse-transcribed with 200 units of SuperScript II reverse transcriptase (Invitrogen, Carlsbad, CA) in a 20 ul reaction for 50 min at 42°C followed by heat-denaturation at 70°C for 15 minutes. Two microliters of the first strand reaction product were amplified in six duplicate reactions using standard PCR conditions (94°C for 1 minute, 63°C for 1 minute and, 72°C for 1 minute for 34 cycles) with sequence specific primers. Individual tubes were removed after 24, 26, 28, 30, 32, and 34 cycles and the concentration of specific PCR product determined using an Agilent 2100 BioAnalyzer and DNA 1000 LabChip (Agilent Technologies, Palo Alto, CA). Each RT-PCR was performed twice for each RNA preparation. Two housekeeping control genes, GAPDH and ribosomal protein L32 (RPL32), were utilized as controls with specific primers producing a 599 bp product for GAPDH and a 233 bp product for RPL32.

### Western blot analysis

Cells were lysed in modified RIPA cell lysis buffer (50 mM Tris-HCl, pH 7.4, 1% NP-40, 1% sodium deoxycholate, 0.15 M NaCl, and 1 mM EDTA) with 1 mM phenylmethylsulfonylfluoride (PMSF), 1 mM sodium orthovanadate, 5 μg/ml leupeptin, 2 μg/ml aprotonin and one Complete Protease Inhibitor Cocktail tablet (Roche Diagnostics Corporation, Indianapolis, IN) per 50 ml of buffer. Whole cell protein extract was used directly for Western blot analysis. Protein concentration was determined using Bio-Rad protein assay kit (BioRad). Twenty micrograms of total protein from each sample was separated on a 10% Tris-glycine polyacrylamide gel and transferred onto a polyvinylidene difluoride membrane (Invitrogen, San Diego, CA). Membranes were blocked for 1 h at room temperature in PBS containing 5% non-fat dry milk and 0.1% Tween-20. The membranes were then incubated overnight at 4°C in primary antibody (anti-CA150, anti-thioredoxin, anti-flotillin and anti-ferritin H chain, BD Biosciences Transduction Laboratories, Lexington, KY and Santa Cruz Biotechnologies, Inc, Santa Cruz, CA) diluted 1:1000 in PBS containing 5% non-fat dry milk and 0.1% Tween-20. The membranes were washed in PBS with 0.1% Tween-20 then incubated for one hour in secondary antibody (goat anti-mouse-HRP and rabbit anti-goat-HRP; Santa Cruz Biotechnology, Inc., Santa Cruz, CA). The blots were washed and the proteins detected using the ECL Plus Western Blotting Kit (Amersham Biosciences UK Limited, Buckinghamshire, UK) and X-MAT AR Film (Eastman Kodak, Rochester, NY).

### Cellular migrations and intracellular calcium mobilization

Jurkat T cell migration was examined using a fluorescence-based Transwell chemotaxis assays as previously described [21,61]. CXCR3-and neo-transfected Jurkat T cells were labeled with 10 μg/ml Hoechst 33342 (Molecular Probes) in cRPMI for 30 min at 37°C, and then treated with chol-BCD as described above. The cells were then resuspended in RPMI with 1% FBS to a concentration of 1 × 10^7^/ml. RPMI (0.6 ml) containing 1% FBS with or without 100 ng/ml SDF-1α was added to the bottom wells of the 24-well plate. Transwell chambers with 5 μm pore filters (Corning CoStar, Acton, MA) were then placed into the wells. Cells (1–3 × 10^6 ^in 100 μl) were then added to the chambers. After 2 h, the migrated cells in the bottom wells were transferred to triplicate wells of a 96-well plate in 150 μl volumes. Hoechst fluorescence was measured on a Fluoroskan Ascent FL fluorescence plate reader (Thermo Labsystems, Franklin, MA) at λ_ex _= 355 nm, and λ_em _= 460 nm. Results are expressed as migration index calculated by subtracting the fluorescence intensity of media alone and comparing the values to the fluorescence intensity (relative number) of cells migrated into the bottom chamber in media alone, which is normalized to a value of 1. Fluorescence values were within the linear range of a standard dilution curve.

CXCR3-transfected Jurkat T cells were loaded with the fluorescent indicator, Fura-2AM (Molecular Probes, Eugene, OR), for 30 min, then washed and resuspended in PBS containing calcium and magnesium at 10^6 ^cells/ml [61]. The ratio of free to bound intracellular calcium was determined by spectrofluorometry by monitoring absorption at 340 nM versus 380 nM and emission at 510 nM. The chemokines, CXCL12α and CXCL10 (Peprotech, Rocky Hill, NJ) were utilized in these experiments at 1 μg/ml.

## Results

### CXCR3-transfected Jurkat T cells migrate and mobilize intracellular calcium in response to CXCL12 and CXCL10

**Figure 1:**
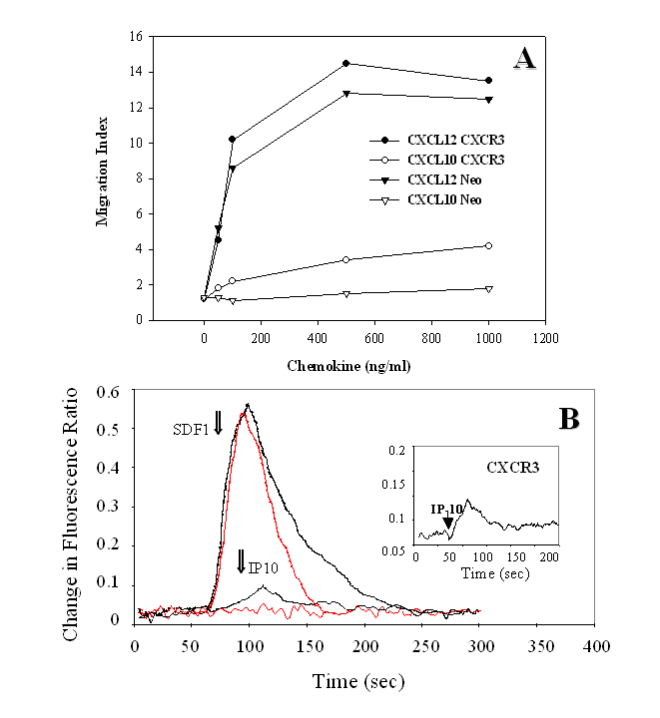
**CXCR3-transfected Jurkat T cells migrate and mobilize calcium in response to CXCL10 and CXCL12. **CXCR3-transfected Jurkat T cells or neo-transfected Jurkat T cells (Panel A) were examined within Transwell chemotaxis chamber for their ability to migrate in response to various concentrations of CXCL10 and CXCL12 as described in the *Methods*. The migration data are expressed as a migration index relative to the number of migrating cells in the absence of chemokine. Panel B shows the mobilization of intracellular calcium within CXCR3-transfected and control Jurkat T cells stimulated with CXCL10 or CXCL12 (1 μg/ml). The data points were collected every 0.48 s and are presented as the relative ratio of fluorescence excited at 340 and 380 nm. Arrows indicate when the chemokine was added to the chambers. The insert within Panel B is a close-up view of the CXCL10 response within CXCR3-transfected Jurkat T cells. We have never observed any calcium mobilization or chemotactic activity by non-transfected or neo-transfected Jurkat T cells in response to CXCL10 (data not shown).

The cells were found to be functionally responsive to both CXCL12 and CXCL10. As shown in Fig. 1A, a subclone of a CXCR3-transfected Jurkat T cell line, which was found by flow cytometry to coexpress both CXCR3 and CXCR4 (Table 1), specifically migrated in response to CXCL12 and CXCL10 in a dose-dependent fashion. Optimal migration for CXCL12 was noted at 0.5–1 μg/ml, while optimal migration for CXCL10 was observed at 1 μg/ml. In contrast, control neomycin phosphotransferase gene-transfected Jurkat T cells only demonstrated responses to CXCL12. Similarly, CXCR3-transfected Jurkat T cells demonstrated a potent calcium mobilization in response to CXCL12 (1 μg/ml) and a modest response to CXCL10 (1 μg/ml), while neo-Jurkat T cells failed to demonstrate any CXCL10 response (Fig. 1B). The modest migration and calcium mobilization observed in response to CXCL10 compared to CXCL12 in this cell line suggests either distinct signaling through CXCR3 or lower cell surface CXCR3 density. Based on the flow cytometric data (Table 1), the mean fluorescence intensity for CXCR4 is approximately 5-fold greater than that for CXCR3 on this transfected cell line. Similar levels of CXCR4 were expressed on all of these cell lines including a neo control or Bcl2-transfected cell Jurkat line. As expected, CXCR2 failed to demonstrate any staining on these cell lines. While there may be differences in CXCR4 and CXCR3 signaling in these cells, differences in receptor density may influence the chemokine-induced gene expression differences described below and thus cannot be ruled out.

### Differential display of mRNA expression in CXCL12-and CXCL10-treated T cells

**Figure 2:**
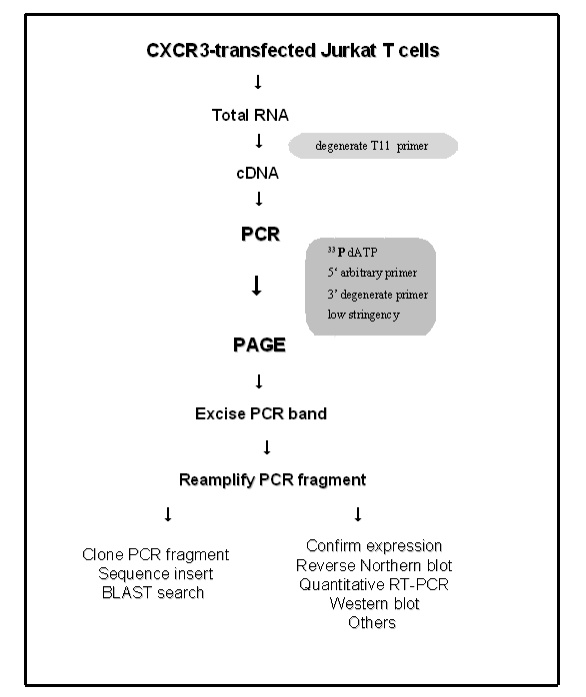
**Flow chart of the RT-PCR-based differential display procedure. **A detailed description of each step is found in the *Methods.*

**Figure 3:**
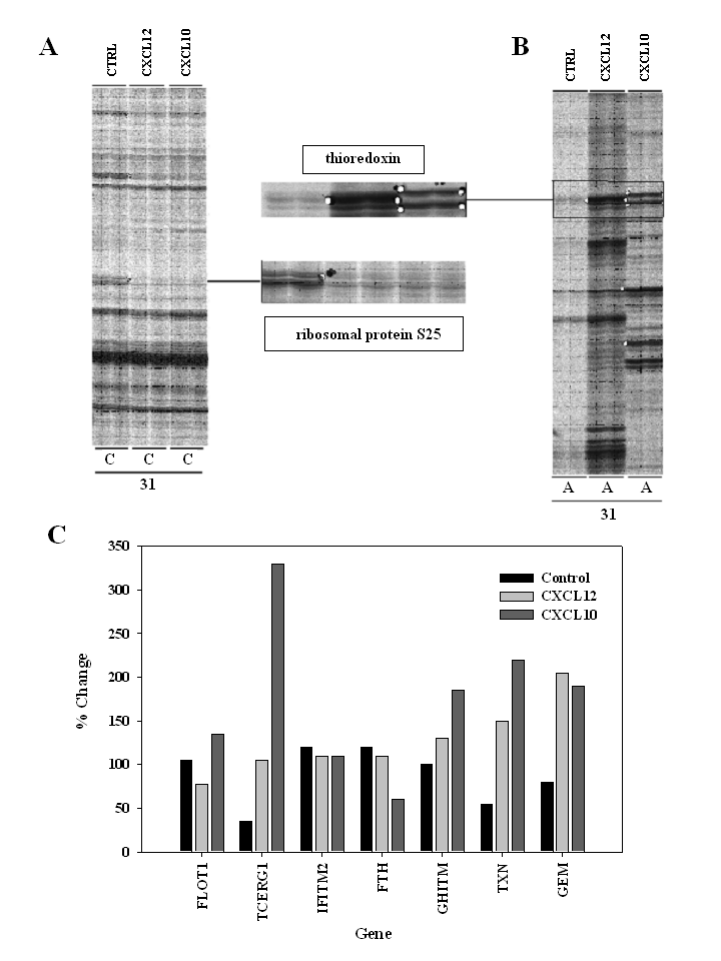
**Differential display autoradiography. **Panel A and B show examples of DDRT-PCR autoradiographs. Chemokine treatments are indicated above and the primer combinations below each cell lane. Panel C shows densitometric measurements of the relative gene expression of individual bands after treatment of the transfected Jurkat T cells with 1 μg/ml of CXCL12 or CXCL10 for 24 hrs.

In an effort to identify genes, which may be upregulated or downregulated by CXCL12 or CXCL10, we examined Jurkat T cells that that expressed endogenous CXCR4 and that had been transfected with CXCR3 using DDRT-PCR analysis (Figure 2, Table 1). Jurkat T cells were stimulated with either CXCL12 or CXCL10 at a concentration of 1 μg/ml for 24 hr. The dose of 1 μg/ml was selected as this concentration yielded optimal migration for both CXCL12 and CXCL10 in the CXCR3-transfected T cells. Total RNA prepared from normal and chemokine-stimulated Jurkat cells were reverse transcribed into cDNA. The resultant cDNAs were amplified with 45 combinations of the arbitrary and oligo(dT) anchored primers. Seventeen cDNA bands were found to be differentially expressed in CXCR3-transfected Jurkat T cells. Fig. 3 shows the representative differential display results obtained with six separate primer combinations. Two cDNA fragments, designated as C31.3 (ribosomal protein S25) and A6.7 (thioredoxin) were identified to be differentially expressed by chemokine treated but not in untreated Jurkat cells (Fig. 3A and 3B). The cDNA fragments were excised from the gel, reamplified, subcloned and sequenced.

### Direct sequencing of DDRT-PCR products

To characterize the sequence identity of DDRT-PCR products, the excised and re-amplified bands were cloned into the PCR-TRAP vector and transformed into GH-competent E. coli. Several colonies were selected to be cultured, plasmids were purified, and the inserts wee subsequently screened by colony-PCR using primers flanking the site of the PCR-TRAP vector. The plasmids containing an insert were sequenced. We successfully sequenced a total of 146 cDNAs. Seventeen mRNAs were differentially expressed post treatment with either CXCL12 or CXCL10, each representing known genes with established functions. Five additional RNAs were also identified defining known genes with unknown functions and nine identified hypothetical or predicted genes of unknown function (Table 2). Twenty-one genes were upregulated in CXCR3-transfected Jurkat cells following both CXCL12 and CXCL10, four genes displayed a discordant response and seven genes were down regulated by both chemokines (Fig 3).

**Table 2:** Differentially expressed genes post chemokine treatment.

BAND	Bp	GENE	LOCUSLINK	GENEACC	NAME
C10.5		AD24	64318	NM_022451	AD24 protein
A09.4		BM-002	51569	NM_016617	BM-002 hypothetical protein
A09.2		DDX1	1653	NM_004939	DEAD/H (Asp-Glu-Ala-Asp/His) box polypeptide 1
C27.3		DDX30	22907	NM_014966	DEAD/H (Asp-Glu-Ala-Asp/His) box polypeptide 30
G04.3		DFKZp564M113	none	AL049282	Homo sapiens, clone MGC:5564, mRNA
A10.3		DFKZp566D193	25847	AL050051	DFKZp566D193 protein
A25.1		EIF4B	1975	NM_001417	eukaryotic translation initiation factor 4B
A28.3		EPB41L2	2037	NM_001431	erythrocyte membrane protein band 4.1-like 2
C10.1		FLJ12876	64767	NM_022754	hypothetical protein FLJ12876
G01.1	101	FLOT1	10211	NM_005803	flotillin 1
G09.2	444	FTH1	2495	NM_002032	ferritin, heavy polypeptide 2
C07.3	329	GHITM	27069	NM_014394	growth hormone inducible transmembrane protein
C10.4		IFITM2	10581	NM_006435	interferon induced transmembrane protein 2 (1–8 D)
A01.2		INVS	27130	NM_014425	inversin
C03.3		KIAA0478	9923	NM_014870	KIAA0478 gene product
G04.6		KIAA0648	23244	AB014548	KIAA0648 gene product
G01.4		KIAA1600	57700	AB046820	KIAA1600 protein
A04.7	401	LOC51053	51053	NM_015895	geminin
C35.1		LOC51633	51633	NM_016023	CGI-77 protein
G35.1		MAP3K10	4294	NM_002446	mitogen-activated protein kinase kinase kinase 10
A27.14		MGC10744	84314	NM_032354	hypothetical protein MGC10744
C15.3		MGC4809	91860	AF308287	serologically defined breast cancer antigen NY-BR-20
A25.3		NCBP1	4686	NM_002486	nuclear cap binding protein subunit 1,80 kD
A27.12		NCBP2	22916	NM_007362	nuclear cap binding protein subunit 2, 20 kD
G11.1		RPL7	6129	NM_000971	ribosomal protein L7
G30.2		RPS12	6206	NM_001016	ribosomal protein S12
C31.3		RPS25	6230	NM_001028	ribosomal protein S25
A11.1		TCERG1	10915	NM_006706	transcription elongation regulator 1 (CA150)
A06.7	157	TXN	7295	NM_003329	thioredoxin
C15.5		VIT1	55519	NM_018693	vitiligo-associated protein VIT-1
G03.6		WBP11	51729	NM_016312	WW domain binding protein 11

**Figure 4:**
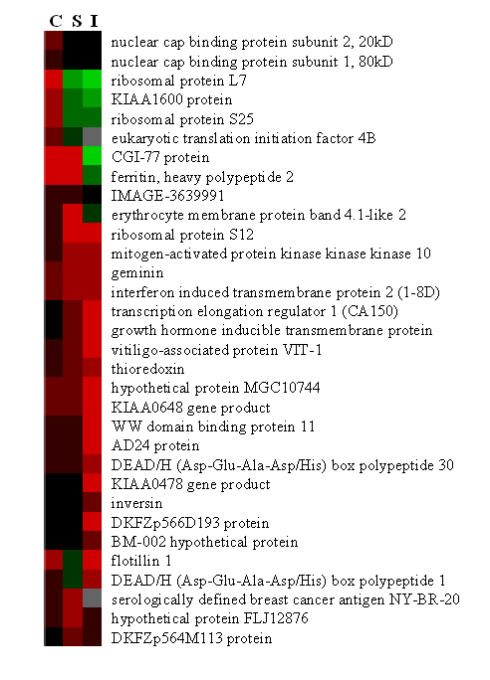
**Comparison of the relative gene expression by CXCL12-versus CXCL10-treated T cells. **Changes in gene expression were displayed using computer programs (Cluster, Tree-View-Eisen) using an arbitrary densitometric scale of 0–4 for all named genes found by DDRT-PCR. Red indicates upregulation, green down-regulation, and black-to-gray means no change. The columns are labeled as C (Vehicle control), S (CXCL12), and I (CXCL10).

Sequence analysis indicated that the 401 bp A4.7 cDNA is highly homologous to the gene LOC51053 that is also known as geminin, a cell cycle regulator. The 157 bp A6.7 cDNA fragment is 94% identical to the 501 bp human thioredoxin (TXN) gene. The sequence of the band A11.1 was homologous to the transcription elongation regulator 1 gene (TCERG1) also known as CA150. The 329 bp C7.3 cDNA fragment is 87% identical to the published human gene encoding the growth hormone inducible transmembrane protein (GHITM). The sequence of the band C10.4 was homologous to the interferon-induced transmembrane protein 2 (IFITM2) gene, a member of the 1–8 gene family whose members are strongly induced by both type I (IFNα, IFNβ) and type II (IFNγ interferons). The 102 bp G1.1 cDNA fragment is 97% identical to the human gene encoding flotillin 1 (FLOT1) that is thought to play a role in vesicular trafficking and signal transduction. Sequence analysis indicated that the 444 bp G9.2 cDNA, as well as several other bands, were highly homologous (>95%) to the gene encoding the iron-storage protein ferritin heavy polypeptide 1 (FTH1). The sequences of the other cDNAs included in Table 2 showed significant homology to published sequences in Genebank. However, it should be noted that in a number of cases, the nucleotide sequence of the cDNA matched named genes about which little is known or matched, in some cases for over 500 bp, the sequence of hypothetical or predicted genes. Several ribosomal proteins including L7, S12 and S25 and ferritin heavy chain (FTH1) were identified several times in our DDRT analysis. In Figure 4, these changes in gene expression as assessed by DDRT are more clearly displayed post cluster analysis using an arbitrary densitometric scale of 0–4 for all named genes found by DDRT-PCR. Red indicates upregulation and green down-regulation in response to chemokine treatment.

### Identification of differentially expressed RNA transcripts associated with CXCL12 or CXCL10 treatment

As a large fraction of DDRT-PCR products have been shown to yield weak or barely detectable signals in the Northern blot analysis, we sought to confirm the identity and differential expression of these bands via RT-PCR analysis. We focused our attention on a subgroup of the seventeen DDRT-PCR products representing known genes with established functions. Gene specific primers were designed to produce unique amplimers between approximately 150 and 500 base pairs in size.

**Figure 5:**
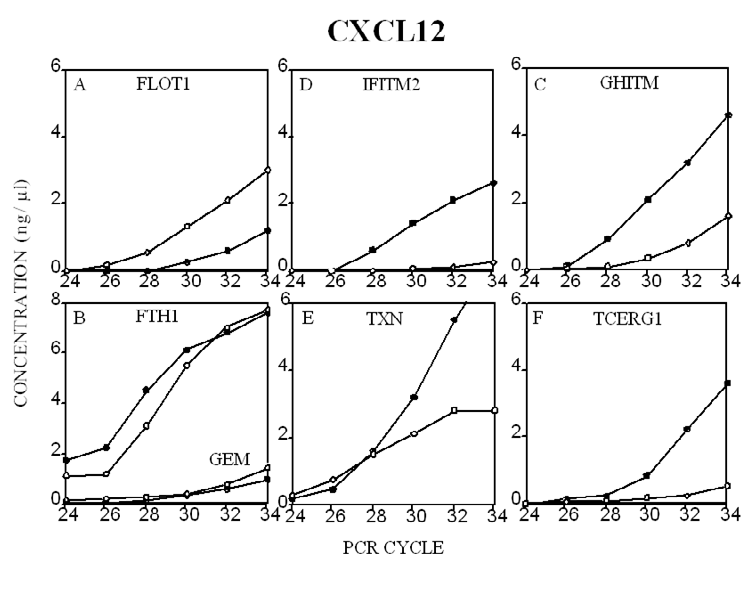
**Quantitative RT-PCR measurement and verification of CXCL12-induced gene expression. **A detailed description of the procedure employed is found in the *Methods*. Each transcript was normalized to the expression of RPL32 in the same PCR reaction.

RT-PCR analysis was performed on the Agilent BioAnalyzer 2100 System. The advantage of using this system to examine competitive PCR products lies in the accurate absolute and relative quantitation of each amplified product. Small differences in the amount of amplimer product, which cannot be detected using slab gel analysis, are more easily analyzed on this equipment permitting RT-PCR to be used to measure changes in gene expression. RNA from CXCR3 receptor-transfected T cells, both before and post treatment with CXCL12 or CXCL10 was reverse transcribed in bulk and aliquots RT product amplified by PCR using specific sets of primers. Initially, a 599 bp GAPDH amplimer was utilized as a housekeeping gene. However, it was noted that GAPDH significantly up-regulated post treatment of the cells with either CXCL12 or CXCL10. To address this issue, a 233 bp ribosomal protein L32 amplimer was utilized as a housekeeping gene for the subsequent comparison of gene expression levels. In Figures 5 (CXCL12) and 6 (CXCL10), each of the seven genes (FLOT1, GEM, GHITM, FTH1, IFITM2, TXN and TCERG1) examined were found to be either up-regulated or down-regulated as predicted by the differential display autoradiogram bands in Fig. 3. However, there was little, if any, relationship between the intensity of the DD band and the quantity of PCR product subsequently detected. These quantitative RT-PCR data confirm our DDRT data and support CXCL12-and CXCL10-mediated gene regulation in the Jurkat line. It should be noted that given that we are utilizing CXCR3-transfected Jurkat T cells in these studies, it is quite possible that differences in CXCR3 and CXCR4 receptor density and signal molecule association may influence the genes induced in response to these chemokines. Moreover, as high doses of chemokine were utilized in the stimulation cultures (1 μg/ml), these supraphysiologic concentrations may also differentially influence the observed differences in gene expression.

**Figure 6:**
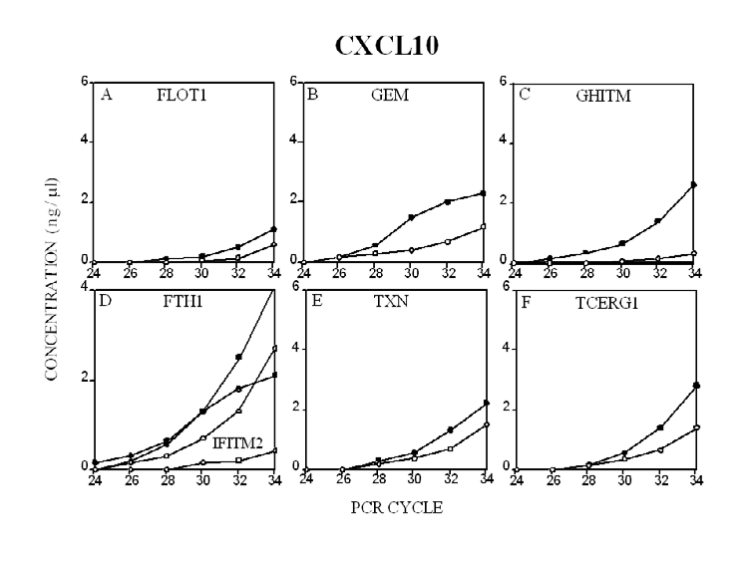
**Quantitative RT-PCR measurement and verification of CXCL10-induced gene expression. **A detailed description of the procedure employed is found in the *Methods*. Each transcript was normalized to the expression of RPL32 in the same PCR reaction.

Additional studies were performed using primary human T cells derived from several different donors to verify the differential CXCL12-and CXCL10-induced gene expression observed in Jurkat T cells. However, despite the clear differences in gene expression in Jurkat T cells after chemokine stimulation for 24 hr in culture, primary resting and anti-CD3/CD28 mAb-activated human T cells demonstrated variable and non-reproducible expression of the geminin, thioredoxin, DDX1, GHITM and TCERG1 gene products (data not shown). This variability may have more to do with the activation state of the primary T cells being utilized and the chemokine receptor expression and density on primary T cells versus the CXCR3-transfected Jurkat T cell subclone. More detailed studies examining CXCL12-induced gene expression in primary human T cells at various stages of activation are the focus of current studies. Moreover, studies using CXCL10 on primary human T cells are quite difficult as the expression of CXCR3 on resting human T cells is quite low to non-existent (>5% on CD3+ T cells with MFI between 5–12) and may be selectively expressed on certain cell subsets. CXCR3 studies in normal human T cells would require an activation of T cells using IL-2 or Th1 polarizing stimuli and thus may not be a valid comparison with the Jurkat T cells utilized in these studies.

### Protein expression in Jurkat T cells post CXCL12 treatment

**Figure 7:**
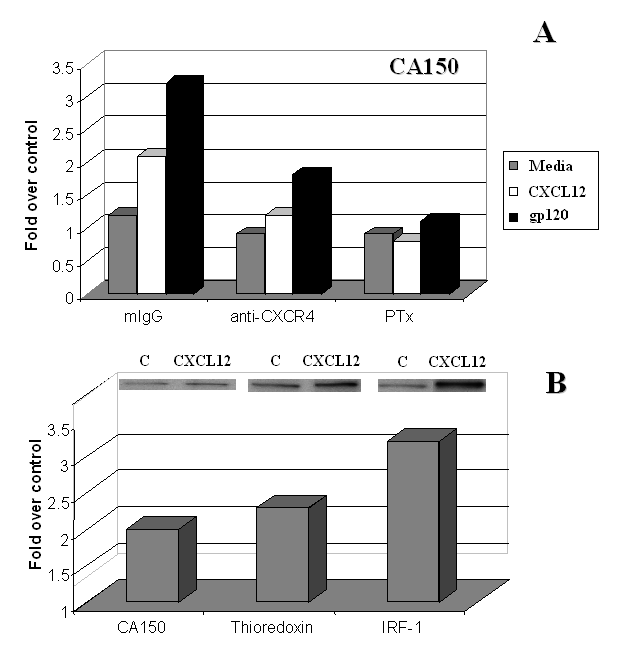
**CA150/TCERG1, thioredoxin and IRF-1 protein expression by Jurkat T cells post CXCL12α treatment. **Jurkat cells were treated for 24 hrs with either CXCL12 (A, B) or gp120 IIIB (A) at 1 μg/ml in the presence or absence of control mouse IgG, mouse anti-human CXCR4 mAb, or pertussis toxin (PTx; 200 ng/ml) at 37°C. After incubation, the cell pellets were isolated, counted, washed, and subsequently lysed with the detergent. Protein determinations were then performed. Samples were loaded at 20 μg per lane on a 10% polyacrylamide gel. After electrophoresis, the gels were transferred using a transfer apparatus to an immobilon membrane and stained for CA150/TCERG1 (A, B), thioredoxin (B) or IRF-1 (B) expression via Western blot analysis (shown in panel B for each of these proteins versus control). The results are expressed as fold change versus control expression (post background subtraction).

To further confirm the expression of several of these genes, Western blot analysis was subsequently performed on total cell lysates of CXCL12-or gp120 IIIB-treated Jurkat T cells. The results shown in Fig. 7A demonstrate that, similar to its gene expression, TCERG1/CA150 levels increased in Jurkat T cells cultured with CXCL12 or the HIV glycoprotein, gp120 IIIB, over a 24 hr time period. This expression was found to be CXCR4-dependent as neutralizing antibody to CXCR4 (but not control mouse IgG) inhibited the CA150 increase in response to CXCL12 and gp120 IIIB. This CA150 increase was inhibited by the addition of pertussis toxin, a Gα1 inhibitor. Given that gp120 IIIB binds to both the CD4 and CXCR4 molecules on the surface of human T cells, the similar results between CXCL12 and gp120 IIIB treatment suggests an active signaling role for gp120 IIIB through CXCR4. Figure 7B demonstrate that TCERG1 (CA150) and thioredoxin were both increased post CXCL12 treatment. In addition, although not identified in our DD studies, the signaling protein, interferon regulatory factor-1 (IRF-1), was also examined on our gels and increased significantly within Jurkat T cells post CXCL12 treatment. Similar to its gene expression, the gene product, flotillin-1, was found to be decreased post CXCL12 treatment in several experiments; however, these results were found to be highly donor variable (data not shown). We believe that this variability was most likely due the use of total cell lysates instead of membrane preparations. In addition, despite examining numerous lysates preparations and blots, we were unable to detect ferritin heavy chain expression by Western blot in any of these studies.

## Discussion

Studies on the alteration of gene expression following chemokine-receptor ligation and the identification of genes that are differentially expressed can provide relevant and important insight into a variety of biological processes and disease etiologies. To date, numerous approaches, model systems, and techniques have been used to search and identify chemokine-modulated genes [28-33]. In the present study, we used the PCR differential display method to screen genes and compare the changes that occur following CXCL12/CXCR4 and CXCL10/CXCR3 ligation. Both the CXCR3 and CXCR4 chemokine receptors are broadly expressed in many tissues and ligation to their specific chemokines is known to result in downstream signaling through several different pathways such as Ras, and PI3 kinase. PI3 kinase and JAK/STAT signaling pathways activated by CXCL12/CXCR4 ligation play roles in lymphocyte chemotaxis in response to these signals [34-37]. Although less well characterized, the interaction of CXCR3 with its ligands, in this case CXCL10, results in increased chemotaxis and activation of the Ras/ERK cascade as well as stimulation of Src phosphorylation and kinase activity and increased activity of phosphatidylinositol 3-kinase and its downstream pathway Akt [38]. Despite knowledge of the activation of multiple cytokine-induced signaling pathways, an understanding of the transcriptional mechanisms whereby CXCR3 and CXCR4 ligation regulates and mediates cellular change remains largely unknown.

In the present study, we have identified 31 cellular genes that were either up-or down-regulated in CXCR3-transfected Jurkat T cells following treatment with either CXCL12α or CXCL10. Suzuki and colleagues [30] have recently examined gene expression in Jurkat T cells treated with 380 ng/ml of CXCL12 (a dose that demonstrated optimal Jurkat migration in their hands) for various time periods up to 12 hours in the presence or absence of serum using cDNA microarray gene analysis. The arrays utilized were 2140 cDNA microarray with 1847 unique genes http://nciarray.nci.nih.gov/cgi-bin/gipo. Many of the genes identified in this study are associated with detoxification, DNA repair, apoptosis, migration, T cell receptor signaling and interferon signaling. While many of the chemokine-induced genes observed in our study are not found on the arrays used by Suzuki et al. [30], we did identify several genes and proteins with similar functional associations as this group, namely interferon-induced transmembrane protein 2 and interferon regulatory factor-1 (interferon-associated genes), thioredoxin and ferritin (detoxification/redox), growth hormone inducible transmembrane protein (apoptosis), flotillin-1 (cell signaling and migration) and geminin (cell signaling/division). It should be noted that we do believe there are differences in the systems utilized by our groups as we failed to confirm using real time RT-PCR and flow cytometry several of the genes identified by Suzuki et al. Differences in the cell lines (bulk line vs. subclone vs. transfected), culture conditions, serum status, dose of chemokine utilized (380 ng/ml vs. 1 μg/ml) and receptor density may account for such disparity. Regardless of these differences, the relationship between the genes identified in our current study and those within the Suzuki study and their role in chemokine biology and function remains to be determined.

Although not previously linked to chemokine receptor-ligand signaling, expression of several of these genes such as interferon-induced transmembrane protein 2 (IFITM2) and growth hormone inducible transmembrane protein (GHITM) are recognized to be a part of the interferon signaling system that includes Janus kinases and their downstream target STAT proteins [39,40]. IFITM2 is a member of the large 1–8 gene family whose members are strongly induced by both type I (IFNα, IFNβ) and type II (IFNγ) interferons [41,42]. However, additional information regarding the molecular function of this protein remains unknown. Likewise, information about the molecular function of GHITM is also quite sparse. GHITM displays some similarity to the testis-encoded transcript (TEGT). While the function of TEGT is also unknown, its amino acid sequence predicts a 26.5 kDa integral membrane protein with seven potential transmembrane domains suggesting a possible receptor function [43]. Interestingly, TEGT is 100% homologous to BAX-inhibitor 1 (BI1) [44,45]. Studies of cells overexpressing BI1 have shown its role as a regulator of cell death pathways controlled by BCL2 and BAX [44]. GHITM has also been referred to by several other names including DERP2, My021, PTD010 and HSPC282. DERP2 is a novel protein originating in human hair papilla cells that has an effect on regulating the growth of hair. The logic behind relating these seemingly inane associations is further supported by knowledge that hair follicle development requires Sonic hedgehog expression [46] that is essential for CXCL12 signaling in the CNS [47,48].

The transcriptional cofactor, CA150, whose gene is now designated transcription elongation regulator 1 (TCERG1) is capable of repressing transcription from many viral and cellular promoters whose initiation is dependent upon the presence of a TATA box [49]. CA150 represses RNA polymerase II (RNAPII) transcription by inhibiting the elongation of transcripts. CA150 is a transcriptional co-activator of HIV-1 Tat and therefore is likely to regulate many cellular genes involved with cell signaling, proliferation and differentiation [50]. A portion of the CA150 molecule contains six FF domains and this region appears to directly bind to the phosphorylated carboxyl-terminal domain of the largest subunit of RNAPII. WW1 and WW2 functional domains are also found in CA150 near the FF domains and appear to fine-tune the repression of transcription through their association with the ubiquitous splicing-transcription factor SF1. At present, CA150 is believed to bind to the phosphorylated C-terminal repeat domain of RNA polymerase II of the elongating RNAPII with SF1 targeting the nascent transcripts [51]. CA150 also has been found by DD to be up-regulated in all-trans retinoic acid (ATRA)-induced apoptosis of H9 and SR-786 T cell lymphoma cell lines [52] and to be significantly increased in striatal and cortical brain tissue from individuals with the neurodegenerative disorder Huntington's disease (HD) [53]. Interestingly, a small subset of HD patients with early onset of symptoms have a mutation in the region of the HD gene that increases its binding to CA150 leading to the suggestion that CA150 may interfere with the transcription of genes essential for neuronal survival. Despite these findings, the role of CA150 in T cell activation and survival is currently unknown.

Geminin (LOC51053) is a 25-kDa protein that inhibits DNA replication and is degraded during the mitotic phase of the cell cycle [54]. This protein has generated considerable interest due to its critical role in replication licensing [55,56]. In order to successfully replicate, eukaryotic cells must assure that their chromosomes are duplicated only once in each cell cycle. A process called "licensing" assures that chromatin can only undergo another round of replication after it has passed through mitosis. Geminin inhibits DNA replication by accumulating during metaphase, binding to and inactivating CDT1 and then undergoing degradation during the metaphase to anaphase transition [54,57]. It is hypothesized that geminin may have evolved to couple S-phase regulation to growth and development signals [55]. As geminin is a powerful negative regulator of the cell cycle, it also may function as a tumor suppressor protein.

Flotillin-1 (FLOT1), also known as Reggie-2, encodes a caveolae-associated, integral membrane protein [58]. Caveolae are small indentations on the plasma membrane that are involved in vesicular trafficking and signal transduction. Purified caveolin-rich membranes are enriched for a variety of lipid-modified signaling molecules such as G proteins, Src family kinases, Ras and nitric oxide synthetase [59] and also are populated with members of several families of integral membrane proteins [60]. Recently, flotillin 1 and flotillin 2 have been recognized to comprise a second family of integral membrane caveolin proteins. The function of these flotillins has not been determined but their expression levels are independent of the other caveolin family members. In at least some cell types, caveolins and flotillins are capable of forming hetero-oligomeric complexes and are believed to play a role in receptor signaling [60].

Movement of CCR5 and CXCR4 molecules into lipid rafts is important in the maintenance of receptor conformation and through this mechanism rafts modulate the binding and function of receptors [61,62]. Flotillin 1 and 2 along with stomatin are the major integral protein components of erythrocyte lipid rafts [63]. Although flotillins are major components of caveolae, these proteins may also be components of lipid rafts in other differentiated cell types such as adipocytes, endothelial cells, fibroblasts and immune cells.

Thioredoxin (TXN) is a 12-kDa oxidoreductase enzyme containing a dithiol-disulfide active site. It possesses a variety of biological functions including the ability to modulate the DNA binding activity of the ligand-activated transcription factor aryl hydrocarbon receptor (AHR) as well as the activity of several other transcription factors including general transcription factor IIIC, NF-κB, and AP-1 [64,65]. Reactive oxygen species generated through cellular metabolism can function as cellular second messengers through the regulation of numerous signal transduction pathways. Thioredoxin protects cells against TNF-induced cytotoxicity, general oxidative stress and is able to scavenge free radicals [66]. As increasing evidence accumulates that oxidative stress plays a crucial role in many age-associated diseases and various neurodegenerative disorders, the importance of regulating and maintaining cellular redox status by intracellular redox-regulating molecules such as thioredoxin becomes important to maintain tissue homeostasis [67-69].

Ferritin is a highly conserved iron-binding protein that in its cytosolic form is composed of 2 subunits, ferritin H and ferritin L, each encoded by a distinct gene. Depending on many factors including tissue type, redox status, and the inflammatory state of a given cell or tissue, the ratio of H to L subunits varies greatly [70]. Several proinflammatory cytokines including TNFa, IL-1a (but not IL-1β, IFNg and IL-6) has been shown to transcriptionally induce ferritin heavy chain (FTH1) expression [71,72]. TNFa regulation of FTH1 is through its binding to the p50 and p65 subunits of NFκB [73]. In addition, expression of FTH1 appears to be regulated by insulin, IGF-1 and thyroid hormone [74,75]. Cytokines also regulate the post-transcriptional modification of ferritin possibly through their ability to induce iNOS [74,75]. As noted by Torti & Torti [70], the pathways that link ferritin gene expression with cell stress and altered growth regulation are just beginning to be explored and based on present knowledge are very complex and multifaceted.

## Conclusions

In the current report, we do not reveal a specific physiological role for the gene expression changes that have been observed by DDRT-PCR post CXCL12 or CXCL10 signaling. As noted above, many of the more fully characterized genes have multiple interactions utilizing a number of distinct signaling pathways (e.g., JAK/STAT, AP-1) that are frequently utilized by other cytokine family members. A more detailed understanding of the various genes differentially induced by chemokines via ligation of their cell surface receptors or during the chemotactic process should provide some insight into the process of cell migration and activation and may identify novel targets for therapeutic intervention.

## Competing interests

None declared

## Authors' contributions

JEN, RJS, LS, DB, VDD, EMS and DDT performed the experiments. JEN and DDT prepared the figures and wrote the paper. DDT also supervised the work and edited the manuscript. All authors have read and approved the final manuscript.

## Abbreviations

differential display, DD; growth hormone inducible transmembrane protein, GHITM; GEM, geminin; transcription elongation regulator 1, TCERG1; thioredoxin, TXN; DEAD/H box polypeptide 1, DDX1
